# Epidemiological Society

**Published:** 1897-04-03

**Authors:** 


### Medical Progress and Hospital Clinics.

[*The Editor will be glad to receive offers of co-operation and contributions from members of the profession. All letters should be addressed to* The Editor, at the Office, 28 & 29, Southampton Street, Strand, London, W.C.]

### EPIDEMIOLOGICAL SOCIETY.

At the last meeting, held on March 19th, a paper was read by Dr. James Niven on "The Prevention of Tuberculosis," a paper which is of considerable interest as affording some indication of the demands likely to be made by sanitarians with the object of checking this disease. After paying a high tribute to the philosophical boldness and masterly style with which Villemin had unfolded the pathological problems of tuberculosis, he proceeded to give a review of our present knowledge in regard to its infective nature, and the means by which it is spread from person to person.

In considering the various adjuvant influences which had been put forward as having to do with the production of tuberculosis, he endeavoured to show that they only predisposed to the disease by facilitating and intensifying the dissemination of the infective bacilli, whether by way of dried sputum, dried bowel discharges in the case of intestinal tuberculosis in children, or from the saprophytic growth of the organism in unhealthy dwellings.

An odd bacillus, he said, would have practically no chance unless it happened to light upon, or be carried by a leucocyte to, an injured spot; hence healthy individuals were not likely to be attacked by tuberculosis unless assailed by a considerable number of infective particles.

This was the keynote to his recommendations in regard to prevention. He did not look upon street dust as infectious, nor did he consider that risk was run even in the presence of invalids unless the sputum or the infected fæces were allowed to dry. Where, however, expectoration was allowed to dry on the floors or on handkerchiefs, and was thus spread, or fæces dried on the body or on clothes, and infected dust was thus allowed to rise into the atmosphere, there the risks of infection were great; and in his view the generally accepted fact that overcrowding predisposed to tuberculosis was to be explained, not by the overcrowding itself being injurious, but by the increased likelihood of there being a consumptive in the crowd, and especially by the much greater probability of dust being raised where many people were congregated together.

After considering the predisposing causes, such as overcrowding, the inhalation of vitiated air, injuries of the respiratory tract resulting from various occupations, and the predisposing influence of certain diseases and bad habits, he maintained, that "in the production of tuberculosis each adjuvant may in its turn be dispensed with, providing the fundamental requirement of infection is present; infection which must be abundant and probably must be prolonged for the healthy, but which may in comparatively small amount affect those who have sustained injury or who are constitutionally feeble."

The drift of all this is, of course, to show that the infection can be got at and controlled. So long as we look upon it as practically omnipresent it may fairly be argued that to fight against it is hopeless; once admit, however, that it only exists, in injurious intensity, in certain places, and the whole aspect of the problem is altered.

In regard to this question of the comparative importance of infection and of predisposing conditions in the etiology of phthisis, we cannot help remarking that the evidence of the frequency with which phthisis is thrown off, quoted by Dr. Niven, may be taken to teach a very different lesson from the one he inculcated. If 39 per cent. of the autopsies at the Manchester Royal Infirmary show evidence of cured phthisis, it may fairly be argued that the infection—the mere catching of the disease—is but a minor influence in producing the high death-rate from consumption, the really important matter being the surrounding conditions of health and mode of life, which, after the patient has received his infection, will decide whether he shall live or die.

Among the preventive measures advocated by Dr. Niven, the first mentioned has to do with diagnosis. He urges the importance of ascertaining the real nature of cases spoken of as "chronic bronchitis," and of cases of tuberculous disease of the intestines, and says that, either by search for the bacilli or by the injection of tuberculin, the nature of such cases should be cleared up "in the interests of others than the patient."

Then comes education. The medical attendant should make it his duty to give well weighed and clear counsel in regard to preventing the sputum becoming dried and diffused as dust. The health authorities should place at the disposal of the practitioner printed instructions, which he might give to his patients, as to how this could best be done. The veterinary surgeon, also, should ascertain the extent to which tuberculosis exists among domestic pets, and must teach farmers how to keep their herds free from the disease. Workpeople should be instructed how those who are working in confined rooms may be protected from the spread of infection, and notices should be posted up "advising that every workman afflicted with a chronic cough should carry a Dettweiler's pocket spittoon, and that they must not spit on the floor or in a pocket-handkerchief," and workpeople afflicted with a chronic cough should be educated not even to carry a pocket-handkerchief. A new commandment should be added to those taught in schools, viz., "Thou shalt not spit." Railway companies must provide against the discharge of sputum in railway carriages. The aid of the clergy must be invoked for the protection of public assemblies; special rules must be devised for the guidance of keepers of hotels, private lodging houses, and hydropathic establishments. The sanitary authorities of all health resorts must be especially vigilant in carrying out measures for the disinfection of rooms which have been occupied by consumptive persons, and, in fact, the whole population must be furnished with brief and simple directions for preventing the communication of phthisis. Elaborate care must be exercised in regard to the keeping of cows, and their constant supervision, and herds should be tested with tuberculin. The public also must be educated to demand a guarantee that the milk supplied is free from tuberculosis as shown by the tuberculin test.

Tuberculosis should also, according to Dr. Niven, be made a compulsorily notifiable disease, and the persons affected must be followed up. The lodging houses must be inspected, and of course disinfected, and then comes the important paragraph, "It is needless to enter on the subject of isolation of cases of tuberculosis at present. When we have once obtained notification of the disease the administrative measures which arise therefrom may be left to develop."

All meat must be inspected, and inspectors must be properly trained to recognise tuberculosis. In regard to milk, the tuberculin test to the cows must be employed; or, in its absence, "samples of every pailful of milk from each farm should be tested for the presence of tubercle bacilli, and the cows should be systematically examined by a veterinary surgeon in every district. This means the establishment of a Veterinary Public Health Service." The medical officer of health should be empowered to order the slaughter of any cow shown to yield tuberculous milk, and powers should be obtained to oblige landlords to construct farm buildings in accordance with the model regulations recommended by the Local Government Board.

This is but a rough sketch of some of the proposals made, from which it may be judged how large is the subject, and how far-reaching are its ramifications when dealt with in the spirit of thoroughness displayed by Dr. Niven.

